# *MYH9* Variant p.(Arg424Gly) Alters Nonmuscle Myosin IIA Contraction, Causing Atypical *MYH9*-related Disease

**DOI:** 10.1016/j.ekir.2026.106343

**Published:** 2026-02-03

**Authors:** Lena Pollinger, Johannes N. Greve, Melanie Grosch, Sara Kaliman, Shada Abuhattum, Martin Kräter, Ina Brauer, Jan T. Schaefer, Francesca Pasutto, Antje Wiesener, Florian J. Wopperer, Cathiana Kolb, Christian H.K. Lehmann, André Hoerning, Christoph Daniel, Kerstin Amann, Jochen Guck, Mario Schiffer, Dietmar J. Manstein, Michael S. Wiesener, Tilman Jobst-Schwan

**Affiliations:** 1 Department of Nephrology and Hypertension, University Hospital Erlangen, Friedrich-Alexander-Universität Erlangen-Nürnberg (FAU), Erlangen, Germany; 2 Institute for Biophysical Chemistry and Structural Biochemistry, Fritz-Hartmann-Centre for Medical Research, Hannover Medical School, Hannover, Germany; 3 Max-Planck-Institute for the Science of Light, Erlangen, Germany; 4 Max-Planck-Zentrum für Physik und Medizin, Erlangen, Germany; 5 Department of Pediatrics and Adolescent Medicine, University Hospital Erlangen, Friedrich-Alexander-Universität Erlangen-Nürnberg (FAU), Erlangen, Germany; 6 Institute of Human Genetics, University Hospital Erlangen, Friedrich-Alexander-Universität Erlangen-Nürnberg (FAU), Erlangen, Germany; 7 Research Center On Rare Kidney Diseases (RECORD), University Hospital Erlangen, Friedrich-Alexander-University Erlangen-Nürnberg (FAU), Erlangen, Germany; 8 Department of Nephrology, Center for rare and genetic kidney diseases, TUM University Hospital rechts der Isar, TUM School of Medicine and Health, Munich, Germany; 9 Department of Dermatology, Laboratory of Dendritic Cell Biology, University Hospital Erlangen, Friedrich-Alexander-Universität Erlangen-Nürnberg (FAU), Erlangen, Germany; 10 FAU Profile Center Immunomedicine (FAU I-MED), Erlangen, Germany; 11 Deutsches Zentrum Immuntherapie (DZI), Erlangen, Germany; 12 Department of Nephropathology, Institute of Pathology, Friedrich-Alexander-Universität Erlangen-Nürnberg (FAU), Erlangen, Germany

**Keywords:** autosomal dominant, glomerulus, kidney disease, MYH9-related disease, proteinuria, thrombocytopenia

## Abstract

**Introduction:**

Pathogenic variants in myosin heavy chain 9 (*MYH9),* encoding the heavy chain of nonmuscle myosin IIA (NMMIIA), cause autosomal-dominant *MYH9*-related disease that may include proteinuric kidney disease, macrothrombocytopenia, cataract, sensorineural deafness, and elevated liver enzymes.

**Methods:**

Whole exome sequencing and segregation analysis were performed in a patient with end-stage renal disease. Histology of kidney and liver biopsies was assessed and blood smears were examined for the presence of Döhle-like bodies. Deformability cytometry and monocyte migration assays were performed. Immortalized podocytes and primary skin fibroblasts of 1 patient were transfected with plasmids containing *MYH9* wild type (WT) or the p.(Arg424Gly) variant. Biochemical studies using recombinantly produced proteins were conducted to assess the variant’s impact on adenosine triphosphate (ATP) turnover and motor function.

**Results:**

We identified the likely pathogenic heterozygous *MYH9* variant c.1270C>G, p.(Arg424Gly) in all affected members of a nonconsanguineous family. Typical microscopic findings, such as Döhle-like bodies or NMMIIA conglomerates were absent. Nonetheless, all patients presented with proteinuric kidney disease, elevated liver enzymes, and intermittent thrombocytopenia. The altered protein showed increased ATP turnover in the presence of actin and enhanced motor activity under both unloaded and loaded conditions.

**Conclusion:**

We identified a novel fully segregating *MYH9* variant causing *MYH9*-related disease. Based on biochemical findings, we report the first gain-of-function variant of *MYH9*. We propose that the enhanced intrinsic motor activity of the p.(Arg424Gly) variant is a key contributor to the disease mechanism. Incorporation of the p.(Arg424Gly) variant into nonmuscle myosin IIA filaments and higher-order actomyosin assemblies may, in principle, affect actomyosin dynamics.

## Graphical abstract

**Figure:**
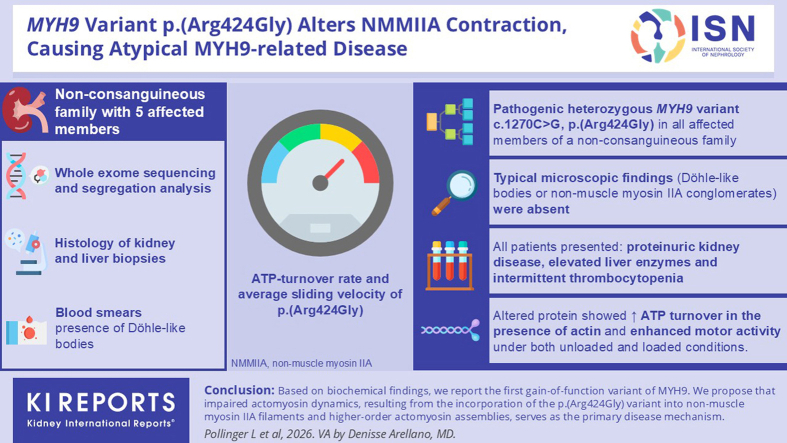

Autosomal dominant pathogenic variants in the *MYH9* gene, encoding NMMIIA, cause a rare monogenic disorder called *MYH9*-related disease (*MYH9*-RD; OMIM# 155100).^1^ It presents variably, from isolated macrothrombocytopenia (May-Hegglin anomaly) to the full Epstein-Fechtner syndrome, including macrothrombocytopenia, proteinuric kidney disease, cataracts, sensorineural deafness, and elevated liver enzymes.^2^ The clinical phenotype varies with genotype, but experimental data clarifying how mutations cause the disease remain limited.

NMMIIA is a large hexameric motor protein composed of 2 myosin heavy chain IIA molecules, each complexed with 1 essential and 1 regulatory light chain. *In vivo*, phosphorylation of the regulatory light chain triggers unfolding of NMMIIA from its inactive 10S conformation into an extended, active state. This conformational change promotes the assembly of individual NMMIIA molecules into short, bipolar filaments capable of interacting with actin (actomyosin), facilitating cellular processes like contraction, migration, and tension maintenance.^3^ Contraction is facilitated by coupling the intrinsic adenosine triphosphatase (ATPase) activity of NMMIIA to structural changes of the molecule, the so-called *powerstroke.*^4^ This precisely tunes mechanochemical coupling and concomitant force output which is of importance for the role of NMMIIA in cell migration,^5^ cell division,^6^ autophagy, and red blood cell morphology.^7^

Reported *MYH9* pathogenic variants are usually associated with congenital platelet macrocytosis and result in abnormal distribution of NMMIIA within granulocytes and other leukocytes forming Döhle-like bodies and NMMIIA aggregates.^8^^,^^9^ NMMIIA is vital for kidney podocytes and for maintaining cytoskeleton integrity and function.^10^^,^^11^ *MYH9* pathogenic variants have been implicated in renal disease phenotypes involving the glomerular structure. *In vitro*, NMMIIA predominantly supports podocyte traction, with minimal contributions from other isoforms.^10^

This study reports a novel *MYH9* variant in 5 family members, presenting with proteinuria, progressive kidney disease and elevated liver enzymes, but without the most common *MYH9*-related symptoms, such as primary thrombocytopenia or NMMIIA aggregates in granulocytes. We characterized the pathogenicity of the variant and its impact on molecular function, carrying out ATP turnover and actin filament motility measurements of the altered NMMIIA protein. Additionally, we performed functional tests in podocytes, primary fibroblasts, and patient derived blood cells showing cell type specific effects of this variant.

## Methods

The detailed descriptions of material and methods are provided in Supplementary Material and Methods.

### Clinical Workup

#### Whole Exome Sequencing

Whole exome sequencing of the index patient’s DNA and processing of the raw data (alignments and variants calls, subsequent filtering, and inspection) was performed as described previously.^12^ All relevant variants were confirmed and analyzed in the affected family members by Sanger sequencing. The variants were interpreted according to the guidelines of the American College of Medical Genetics and Genomics^13^ and the combined annotation dependent depletion score.^14^

#### Periodic Acid-Schiff Staining

Kidney biopsies were fixed and embedded in paraffin. Sections were stained with periodic acid-Schiff.

#### Electron Microscopy

Kidney biopsies were formalin fixed, treated with osmium tetroxide and stained with uranyl acetate before embedding in epoxy araldite resin. Sections were rinsed in lead citrate buffer, and analyzed on a transmission electron microscope.

#### Blood Smear Staining

Pappenheim staining of patients’ blood smears was performed. Immunofluorescence stainings were performed with anti-NMMIIA antibody and 4′,6-diamidino-2-phenylindole.

### *In Vitro* Analysis of Contraction Velocity

#### Recombinant NMMIIA Expression and Purification

The generation of the pFastBac1 vectors encoding the NMMIIA-heavy meromyosin-like (HMM) fragment (1-1337 aa) (Uniprot-ID: P35579) fused to an Avi-His_8_-tag and the NMMIIA-2R motor domain construct (1-777 aa) carrying a C-terminal His_8_-tag was previously described.^15^ Vectors carrying NMMIIA-HMM variants and NMMIIA-2R variants were generated by site-directed mutagenesis. Success of site-directed mutagenesis was verified by sequencing.

NMMIIA-HMM WT/variant, NMMIIA-2R WT/variant, and the β-actin-thymosin β4-His_6_ fusion protein were produced in the *Spodoptera frugiperda* (Sf9) expression system. Sf9 cells were infected 1:50 with the corresponding virus. After 72 hours, the cells were pelleted and stored.

NMMIIA-HMM WT and mutant proteins were purified using Ni-Nitrilotriacetic acid affinity-chromatography followed by size exclusion chromatography, as previously described.^16^^,^^17^ NMMIIA-2R WT and mutant proteins were purified as previously described for NMMIIC.^18^ Recombinant human β-actin was purified as previously described.^19^ ɑ-skeletal actin was purified from chicken pectoralis major muscle, as previously described for rabbit ɑ-skeletal actin.^20^

#### Steady-State ATPase Assay

Basal and actin-activated steady-state ATPase of NMMIIA-HMM WT and p.(Arg424Gly) was measured using a reduced nicotinamide adenine dinucleotide -coupled enzymatic assay. Change in absorbance at 340 nm because of oxidation of reduced nicotinamide adenine dinucleotide was recorded. ATP turnover of myosin at the individual actin concentrations was determined by fitting linear equations to the primary data.

#### Unloaded and Loaded In Vitro Motility Assay

The productive interaction between NMMIIA-HMM WT or mutants and F-actin was analyzed in the *in vitro* motility assay.^21^

#### Myosin Single-Turnover Experiments

The effect of p.(Arg424Gly) on ATP binding, hydrolysis, and adenosine diphosphate (ADP) release was investigated by performing single-turnover experiments with the fluorescent ATP-analogue *mant*-ATP (2′-/3′-O-(N′-methylanthraniloyl)-ATP).^22^

### Statistical Analysis and Visualization

Statistical analysis and visualization were performed using GraphPad Prism (version 10, GraphPad Software, Boston, MA) and Origin 2023 (version 2023, OriginLab Corporation, MA). Comparison between groups was performed using Kruskal-Wallis tests or 2-sample *t*-test. The *P* values were defined as follows: ∗ = *P* < 0.05; ∗∗ = *P* < 0.01; ∗∗∗ = *P* < 0.001.

Assessment and visualization of potential implications of variant p.(Arg424Gly) for the NMMIIA structure was performed using ChimeraX (version 1.9, Resource for Biocomputing, Visualization, and Informatics at the University of California, San Francisco).^23^

### Cell Culture

#### Generation of Primary Fibroblasts

Human dermal fibroblasts were generated from sterile skin punches. Control fibroblasts were obtained from a healthy volunteer (sex-matched). Fibroblasts from passages 5-9 were used for experiments.

#### Immunofluorescence Staining and Analysis

Primary fibroblasts were cultured to full density on glass coverslips, followed by immunofluorescence staining using antibodies against NMMIIA and vinculin. Cell size and focal adhesions were analyzed using ImageJ (National Institute of Health).

#### Wound Healing Assays

Primary fibroblasts were seeded on glass coverslips in 24 well plates (scratch assay) or in 4 well plates inside 2 well ibidi wound healing inserts and grown to confluency. Subsequently, the cell layer was scratched in a straight line or the inserts were removed gently. After that, cells were washed and cultured in low serum medium until imaging (0 and 12 hours post scratching/removal).

#### Autophagy Assay

Primary fibroblasts were seeded in 6 well plates and grown to full density. At confluence, the cells were starved for 24 hours or maintained in growth medium, before treatment with 50 μm bafilomycin in dimethylsulfoxide or dimethylsulfoxide for additional 24 hours. Afterwards, cell protein was extracted and Western blotting was performed. Blots were stained using antilight chain 3 beta (LC3b) antibody.

#### Cell Transfection

Plasmid pMYH9-WT-V5 was used as template for site-directed mutagenesis (c.1270C>G, p.(Arg424Gly)). Success of mutagenesis was confirmed by next generation sequencing.

Primary fibroblasts were grown to 80% density on glass coverslips and transfected with plasmids containing either WT or mutated NMMIIA. After 48 hours, cells were fixed and stained immunofluorescently using antiNMMIIA antibody, antiV5, and DAPI.

Immortalized human podocytes were seeded in cell culture flasks and differentiated for 10 days at 37 °C. They were transfected and put on glass coverslips. After 48 hours, the cells were fixed and stained immunofluorescently using an antiNMMIIA antibody, antiV5, and DAPI.

### Blood Cell Analysis

#### Deformability Cytometry

Deformability cytometry measurements of blood were performed as described previously using an AcCellerator instrument (Zellmechanik Dresden).^24^ Protocol changes are described in the supplemental methods. A total of 30 healthy volunteers (f = 11 [mean age in years 37.45 +/−15.08]; m = 19 [mean age in years 34.16 +/−15.95]) were included as volunteers. For the full dataset see: https://dcor.mpl.mpg.de/group/myh9_data

#### Monocyte Migration Assay

Patient monocytes were isolated from blood samples of patients and age- and sex-matched controls (1 per patient). Boyden chamber chemotaxis assay was performed immediately using 100 ng/ml lipopolysaccharide as a stimulant. After 4 hours of migration, cell counts were measured fluorimetrically.

## Results

### Clinical Data and Whole Exome Sequencing

**Figure 1:**
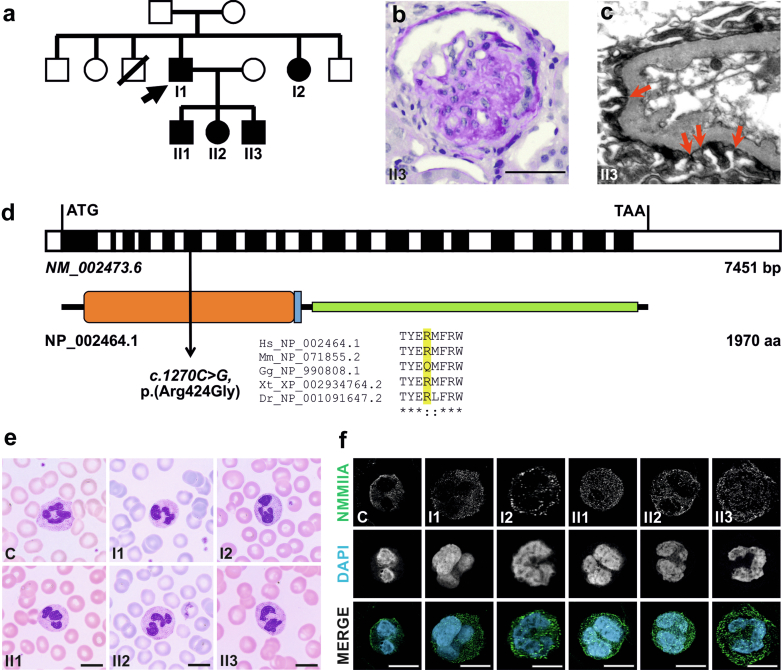
5 affected family members carrying the *MYH9* variant *c.1270C>G*, p.(Arg424Gly) developed FSGS, but do not show Döhle-like bodies and NMMIIA aggregates in granulocytes. (a) All 5 affected family members carry the variant in a heterozygous state. In a kidney biopsy of II3, (b) Periodic acid-Schiff staining shows focal segmental glomerulosclerosis (Scale bar: 50 μm), whereas (c) electron microscopy shows focal podocyte foot process effacement (residual slit diaphragms are marked by red arrows). (d) The variant is located in the exon sequence coding the motor domain (orange bar) of NMMIIA (NP_002464.1). (e) Pappenheim’s stainings of blood smears show granulocytes lacking Döhle-like bodies (Scale bar: 10 μm). (f) Confocal fluorescence microscopy of blood smears shows granulocytes expressing NMMIIA evenly distributed inside the cytoplasm. Blood smears were stained for NMMIIA (green) and DAPI (nuclei; cyan) (Scale bar: 10 μm). FSGS, focal segmental glomerulosclerosis; NMMIIA, nonmuscle myosin IIA.

A nonconsanguineous family of Albanian origin with 5 affected members (Figure 1a) presented with proteinuria, chronic kidney disease, and rapid progression to end-stage renal disease in both adult siblings. Kidney biopsies of I1, I2 (external), and II3 (in our university hospital, Figure 1b and c) revealed mainly focal segmental glomerulosclerosis. The latter showed partially reduced thickness of the basal membrane but no focal thickening or focal splitting as seen before.^2^ I1 and I2 developed pre-senile cataracts. I1, I2, II1, and II2 had sensorineural hearing impairment whereas II3’s hearing status remains uncertain. All had elevated liver enzymes, though liver biopsies in 2 patients (I1, II3) showed normal histology (data not shown). Temporary and moderate thrombocytopenia occurred in I1, I2, and II2 without bleeding events. Table 1 summarizes genetic details and patient phenotypes. Whole exome sequencing of the index patient identified a novel heterozygous *MYH9* missense variant *c.1270C>G*, p.(Arg424Gly), located inside the motor domain of NMMIIA, which is conserved in vertebrates (Figure 1d). The variant segregates in all affected family members (Supplementary Figure S1), but was absent from gnomAD^25^ and the local cohort. Based on 3 different prediction tools (PPH2, SIFT, and MutationTaster),^26^, ^27^, ^28^ the variant was predicted to be deleterious with a combined annotation dependent depletion score^14^ of 22. Additionally, in ClinVar Database a change at the same amino acid (p.Arg424Gln) is already reported as likely pathogenic. According to American College of Medical Genetics and Genomics guidelines (PM1, PM2, PM5, PP3, PP1)^13^ the variant was classified as likely pathogenic (American College of Medical Genetics and Genomics class 4).

**Table 1:** Genetic and clinical data of the 5 affected members of a family with the novel MYH9 variant c.1270C>G, p.(Arg424Gly)

Patient	I1	I2	II1	II2	II3
Gene	*MYH9*				
Nucleotide change	c.1270C>G				
Amino acid change	p.(Arg424Gly)				
Exon	12				
Zygosity	het	het	het	het	het
PPH2 score	0.627				
SIFT	deleterious				
Mut taster	disease-causing				
Amino acid conservation to species	*Danio rerio*				
Gnomad allele frequencies‍ (hom/hemi/het/wt)	n/a				
ACMG classification	4 (not reported previously) (PM1, PM2, PM5, PP1, PP3)				
Gender	male	female	male	female	male
Ethnic origin	Albanian				
Parental consanguinity	no				
Age of onset (ESRD), yrs	childhood (11)	30 (38)	17 (n/a)	18 (n/a)	childhood (n/a)
Kidney phenotype on biopsy	FSGS	MPGN/FSGS	n/a	n/a	MPGN/FSGS
Liver phenotype on biopsy	normal liver tissue	n/a	n/a	n/a	subtle uncharacteristic changes
Platelet count	normal	normal	normal (lower limit)	reduced	normal (lower limit)
Platelet volume	elevated (13.4 fl)	elevated (12.5 fl)	elevated (11.5 fl)	elevated (13.4 fl)	elevated (11.5 fl)
Bleeding events	no	no	no	no	no
Proteinuria	yes	yes	yes	yes	yes
Elevated liver enzymes	yes (transaminases, γ-GT)	yes (γ-GT)	yes (transaminases, γ-GT)	yes (transaminases)	yes (transaminases, γ-GT)
Eye phenotype	cataract	cataract	no	corneal dystrophy	no
Sensorineural hearing impairment	yes	yes	yes	yes	no

ACMG, American College of Medical Genetics and Genomics; ESRD, end-stage renal disease; FSGS, focal segmental glomerulosclerosis; γ-GT, gamma-glutamyl transferase; hemi, hemizygous; het, heterozygous; hom, homozygous; MPGN, membranoproliferative glomerulonephritis; n/a, not applicable; PPH2 score, humvar PolyPhen2 prediction score; SIFT, sorting intolerant from tolerant; wt, wild type; yrs, years.

The American College of Medical Genetics and Genomics classification^13^: PM1 Located in a mutational hot spot and/or critical and well-established functional domain (e.g., active site of an enzyme) without benign variation; PM2 Absent from controls in Exome Sequencing Project, 1000 Genomes Project, or Exome Aggregation Consortium; PM5 Novel missense change at an amino acid residue where a different missense change determined to be pathogenic has been seen before; PP1 Cosegregation with disease in multiple affected family members in a gene definitively known to cause the disease; PP3 Multiple lines of computational evidence support a deleterious effect on the gene or gene product (conservation, evolutionary, splicing impact, etc.)

#### Patients Carrying the p.(Arg424Gly) Variant Do Not Show Döhle-like Bodies

Blood smears from all 5 affected members showed no giant thrombocytes and no Döhle-like bodies or typical NMMIIA aggregates using Pappenheim’s and immunofluorescence staining (Figure 1e and f), indicating absence of typical *MYH9*-related blood cell phenotypes.

#### The p.(Arg424Gly) Variant is Located in the Highly Conserved Motor Domain O-Helix

**Figure 2:**
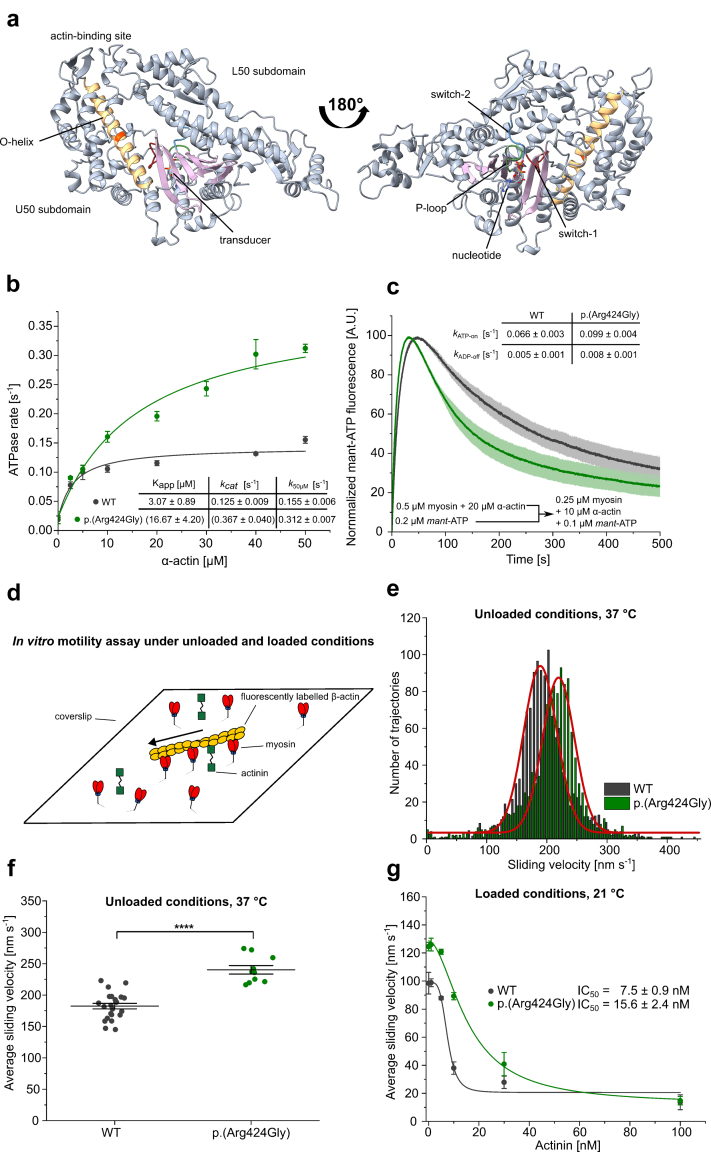
The ATP-turnover rate and average sliding velocity of p.(Arg424Gly) are elevated compared with WT NMMIIA. (a) Structural model of the NMMIIA motor domain with bound ADP generated with AlphaFold 3. Relevant structural elements are highlighted in color and annotated with text. The position of the mutated residue R424 in the O-helix is shown in red. (b) The basal and actin-activated ATPase activity of p.(Arg424Gly) and WT NMMIIA-HMM were analyzed using a reduced nicotinamide adenine dinucleotide-coupled enzymatic assay. The data for the actin-activated ATPase activity is best described by a hyperbolic fit that yields K_app_ (apparent K_m_ for actin) and *k*_ca__t_ (maximal turnover). *k*_50μM_ is the ATPase rate at 50 μM F-actin. Data is shown as the mean ± SD of 3 individual experiments. (c) ATP-binding (*k*_ATP-on_) and ADP-release (*k*_ADP-off_) were analyzed by means of single-turnover experiments with single-headed NMMIIA-2R constructs (p.(Arg424Gly)/WT) and the fluorescent ATP analogue mant-ATP. The initial increase in fluorescence corresponds to mant*-*ATP binding, whereas the subsequent decay in fluorescence is the result of mant-ADP release from myosin. (d) Shown is a schematic drawing of a coverslip with surface-immobilized NMMIIA-HMM molecules, α-actinin molecules and TRITC-phalloidin-labelled actin filaments interacting with the myosin motors. For assays under unloaded conditions, surface coating with α-actinin was omitted. The interaction of surface–immobilized p.(Arg424Gly) and WT NMMIIA–HMM with actin filaments was analyzed using the unloaded and loaded in vitro motility assays. (e) Representative velocity distributions obtained from recorded trajectories of actin filaments on surfaces coated with p.(Arg424Gly) or WT NMMIIA-HMM in an unloaded in vitro motility experiment. The average sliding velocity of the filaments in each experiment was determined by applying a Gaussian fit (red line) to the obtained velocity distributions. (f) Secondary plot of all measured sliding velocities under unloaded conditions. Each data point represents a single experiment (*N* = 23 for WT NMMIIA-HMM, *N* = 10 for p.(Arg424Gly)) in which the sliding velocities of a minimum of 600 filaments have been analyzed. The average sliding velocity in each experiment was determined as described in (e). Data is shown as the mean ± SEM. (g) The motor activity of p.(Arg424Gly) and WT NMMIIA-HMM under load was analyzed using in vitro motility assays in which the surface density of α-actinin was titrated while the surface density of the respective myosin motors was kept constant (frictional load assay). Actin filament trajectories were recorded and evaluated in the same way as in experiments under unloaded conditions. Data is shown as the mean ± SD of 3 individual experiments. A dose-response curve was fitted to the complete data sets (see Supplementary Materials and Methods), which yields the concentration of α-actinin that results in half-maximal inhibition (IC_50_) (*R*^2^ = 0.97 for WT, *R*^2^ = 0.98 for the mutant). Experiments in (b) and (c) were conducted with 1 preparation each of NMMIIA-HMM WT/p.(Arg424Gly) and NMMIIA-2R WT/p.(Arg424Gly). Experiments in D-G were conducted using 2 preparations each of NMMIIA-HMM WT and p.(Arg424Gly). ATP, adenosine triphosphate; HMM, heavy meromyosin-like; NMMIIA, nonmuscle myosin IIA; WT, wild type.

To assess the impact of the p.(Arg424Gly) variant on NMMIIA function, we modeled the NMMIIA motor domain using AlphaFold3 (Figure 2a).^29^ Residue Arg424 in NMMIIA is part of the O-helix (a specific α-helix within the motor domain) in the upper 50 kDa (U50) subdomain of the myosin motor domain, which contributes to the conformational coupling between the actin-binding site, the nucleotide-binding site, and the lever arm region.^4^^,^^30^ Specifically, the O-helix forms a structural hub connecting the actin-binding site via a short surface loop to the central β-sheet of the myosin motor domain, the so-called transducer, which is directly connected to the nucleotide-binding site via switch-1, switch-2, and the P-loop. These structural elements are highly conserved across all human myosin isoforms and sequence variations were shown to contribute to fine-tuning of the functional properties of the myosin motor domain.^31^

Multiple-sequence alignment of human nonmuscle myosin isoforms shows that Arg424 in NMMIIA is part of a highly conserved sequence region in the O-helix (Figure 2a) and is present in 16 out of 27 nonmuscle myosin isoforms (Supplementary Figure S2). The resulting deviation from an ideal α-helix geometry by introduction of a glycine residue at position 424 may alter the strength of the coupling between the actin- and the nucleotide-binding sites.

#### p.(Arg424Gly) Increases Actin-Activated ATP Turnover of NMMIIA

To assess the functional consequences of the p.(Arg424Gly) variant, we produced functional human NMMIIA WT and p.(Arg424Gly) variant proteins in the baculovirus/*Spodoptera frugiperda* protein expression system and purified the proteins to homogeneity. We used heavy meromyosin-like (HMM) fragments of NMMIIA WT and p.(Arg424Gly) and constitutively active, single-headed NMMIIA-2R^32^ constructs as required. Purification of HMM fragments and NMMIIA-2R constructs yielded comparable quantities for both the WT and p.(Arg424Gly) variants, with typical yields of approximately 0.5 mg for HMM and 3 mg for the 2R construct from 2 × 10^9^ cells. For a subset of experiments requiring large amounts of actin, tissue-purified α-actin was employed due to its greater availability, whereas recombinant human cytoskeletal β-actin was used for all assays that directly assess myosin motor function.

Measuring basal and actin-activated ATPase activity of NMMIIA-HMM p.(Arg424Gly) and WT over a range of increasing actin concentrations using a reduced nicotinamide adenine dinucleotide -coupled assay (Figure 2b), we observed no significant difference of the basal ATPase activity between the mutant and WT constructs. In presence of increasing concentrations of actin, we found a significantly stronger activation of the p.(Arg424Gly) ATPase compared with the ATPase of the WT protein. Since the ATPase activity of the p.(Arg424Gly) variant did not reach saturation within the experimentally accessible range of actin concentrations, the resulting *k*_cat_ and K_app_ values should be interpreted with caution. Nevertheless, a comparison of ATPase rates at the highest accessible actin concentration (*k*_50μM_) reveals that the maximal activity of p.(Arg424Gly) is at least 2-fold greater than that of the WT protein (Figure 2b).

Single-turnover experiments with p.(Arg424Gly) and WT NMMIIA-2R constructs, using the fluorescent ATP analogue *mant*-ATP at 10 μm actin (Figure 2c) demonstrated that the p.(Arg424Gly) variant exhibited a 1.5-fold increase in the rate of ATP-binding (*k*_ATP-on_) and a 1.6-fold increase in the rate of ADP-release (*k*_ADP-off_) relative to the WT protein. Analysis of the full width at half maximum of the reaction traces further indicated a 1.7-fold faster overall ATP turnover for the variant.

#### p.(Arg424Gly) Variant Displays Increased Motor Activity

We performed *in vitro* actin filament motility assays with surface-immobilized NMMIIA-HMM p.(Arg424Gly) and WT protein and TRITC-phalloidin labelled actin filaments using epifluorescence microscopy to assess how the observed changes in the enzymatic activity of the p.(Arg424Gly) variant are reflected in its motor activity (Figure 2d).^21^

Under unloaded conditions, with only NMMIIIA-HMM immobilized on the surface, NMMIIA-HMM WT moves actin filaments at an average velocity of 182.4 ±4.4 nm/s. The p.(Arg424Gly) variant moves the actin filaments 32 % faster, with an average velocity of 240.2±6.8 nm/s (Figure 2e and f).

*In vivo*, NMMIIA molecules integrate into contractile networks composed of actin filaments, myosin molecules, and other actin-binding proteins, all subjected to constant external load.^3^ Therefore, we investigated the motor activity of NMMIIA-HMM p.(Arg424Gly) and WT under external load by additionally immobilizing increasing concentrations of the actin-binding protein α-actinin on the surface to generate a drag force (Figure 2d). In experiments with the p.(Arg424Gly) variant, we observed a faster sliding velocity of the actin filament at all α-actinin concentrations up to 100 nm, compared with WT. Fitting a dose-response curve to the data revealed a 2-fold increase in the α-actinin concentration required for half-maximal inhibition (IC_50_), along with a reduced steepness of the curve, both indicative of less efficient inhibition of myosin-driven actin filament sliding by α-actinin for the p.(Arg424Gly) variant. This indicates that the enhanced motor activity of p.(Arg424Gly) persists even under physiological loads, as encountered *in vivo* (Figure 2g). In contrast, we measured reduced sliding velocity of varying degree for the known pathogenic variants p.Arg702Cys^33^^,^^34^ and p.Arg1162Ser^35^ (Supplementary Figure S3).

It is important to note that the mutation’s effect on the mechanochemical function of NMMIIA was consistently observed across both constructs used (HMM and 2R). Analysis of motor activity with 2 independent HMM preparations further confirmed this effect, as both preparations reproducibly showed increased actin filament sliding velocity. Although the other assays were performed with a single preparation each, the reproducibility observed in the motor assays and across constructs provides strong confidence in the validity of the findings.

### Primary Fibroblasts Show Normal NMMIIA Distribution and WT-like Migratory Behavior

**Figure 3:**
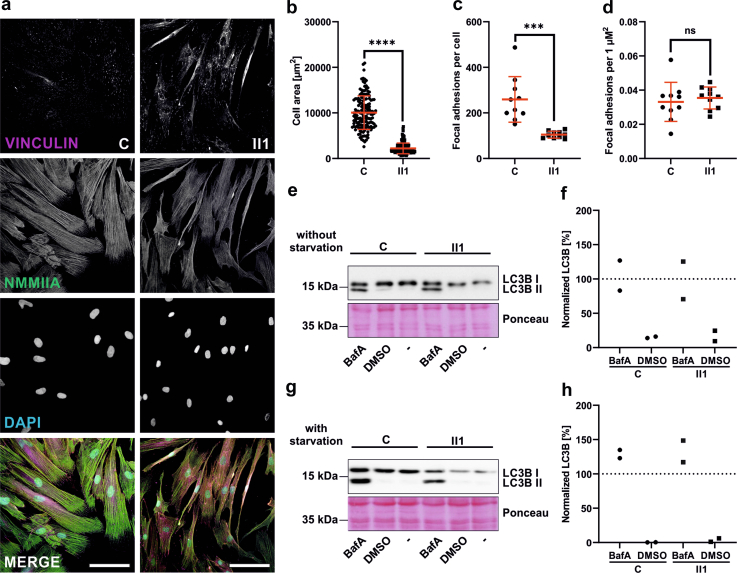
Patient derived *MYH9* c.1270C>G, p.(Arg424Gly) fibroblasts are smaller with an elongated morphology but show no difference in focal adhesion count or autophagy-competency. (a) Immunofluorescence images of fibroblasts cultivated on glass cover slips. Fibroblasts were stained for Vinculin (magenta, focal adhesions), DAPI (cyan, nuclei) and NMMIIA (green), which shows even distribution across the cytoplasm (Scale bar: 100 μm). (b) Cell area measurement of fibroblasts was done using ImageJ. Graph represents mean ± SD. Mann-Whitney test was used to compare patient and control fibroblasts. 50 cells per type were analyzed using ImageJ. ∗∗∗∗*P* ≤ 0.0001. (c) Number of focal adhesions per cell. Mann-Whitney test was used to compare control and patient fibroblasts. 10 cells per type were analyzed using ImageJ. ∗∗∗*P* ≤ 0.001. (d) Number of focal adhesions per 1 μm^2^. Mann-Whitney test was used to compare control and patient fibroblasts. 10 cells per type were analyzed using ImageJ. (e) Western Blot of fibroblast lysates after 24 h bafilomycin A (BafA) or dimethylsulfoxide (vehicle) treatment without a prior starvation step. PVDF membrane was stained for Ponceau (loading control) and LC3B. (f) Densitometric analysis of LC3B bands from (e). LC3B I and II were normalized to Ponceau which corresponds to 100%. Graph shows the ratio between LC3B II and I. (g) Western Blot of fibroblast lysates after 24h bafilomycin A (BafA) or dimethylsulfoxide (vehicle) treatment with a prior 24h starvation step. PVDF membrane was stained for Ponceau (loading control) and LC3B. (h) Densitometric analysis of LC3B bands from (g). LC3B I and II were normalized to Ponceau which corresponds to 100%. Graph shows the ratio between LC3B II and I.

Several pathogenic variants that primarily impact podocytes have been tested in fibroblasts to study the functional consequences, particularly regarding actin dynamics and cellular morphology.^36^^,^^37^ Patient-derived primary fibroblasts obtained from skin punch biopsies offer significant advantages as a cell culture model. These cells express the patient’s pathogenic variants and can be rapidly cultivated, compared with transfected immortalized podocytes. Primary fibroblasts from II1 showed elongated morphology and reduced cell area but no difference in intracellular NMMIIA distribution compared with a healthy control (Figure 3a and b).

Wound healing assays revealed no differences in migration speed (Supplementary Figure S4) or migratory morphology compared with controls. Immunofluorescence staining of focal adhesions using the focal adhesions marker vinculin revealed no significant differences in the focal adhesion density normalized to the cell area (Figure 3a, c, and d).

Pathogenic variants in NMMIIA can disrupt the actomyosin network, leading to impaired autophagic activity.^38^^,^^39^ Autophagy analysis with bafilomycin A, an inhibitor of autophagosomal fusion to lysosomes, showed no differences in LC3B II levels, indicating normal autophagic activity in patient fibroblasts (Figure 3e–h).

### Overexpression of the p.(Arg424Gly) Variant Results in NMMIIA Aggregates in Podocytes but not in Fibroblasts

**Figure 4:**
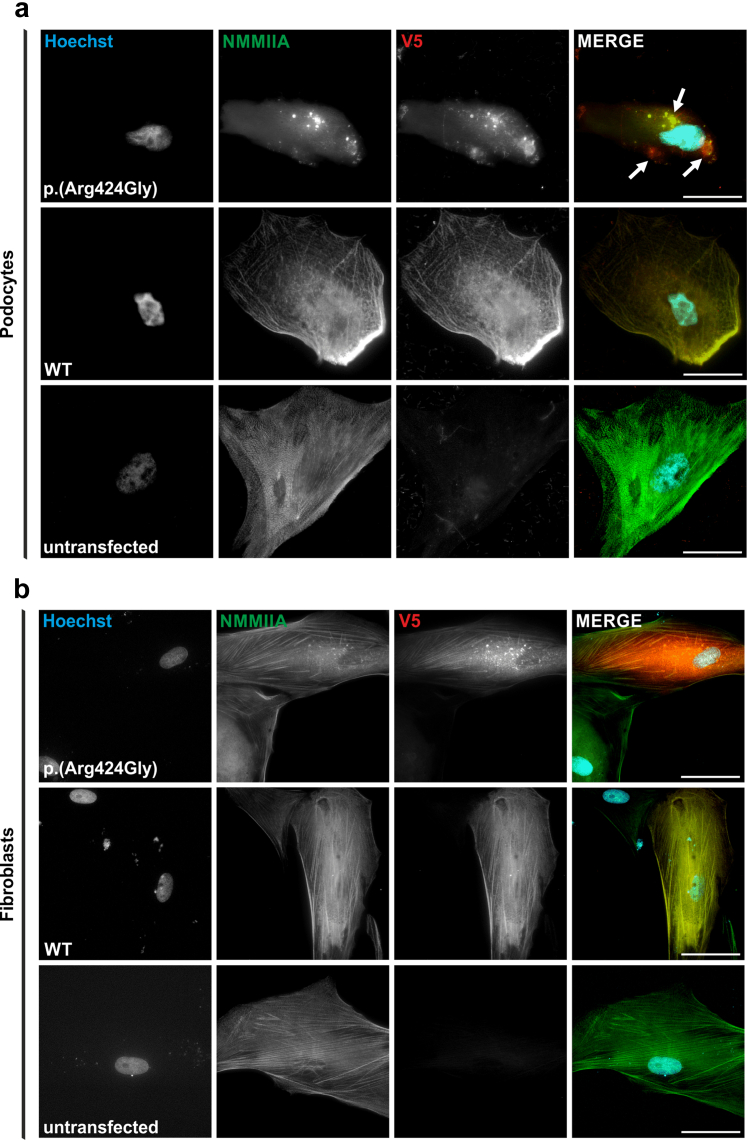
Immortalized human podocytes transfected with p.(Arg424Gly) show prominent NMMIIA aggregates while transfected fibroblasts do not. Immunofluorescence images of podocytes (a) or fibroblasts (b) transfected with pMYH9-WT-V5 or pMYH9-Arg424Gly-V5. Cells were cultivated on glass cover slips and stained for NMMIIA (green), V5 (red) and Hoechst (cyan, nuclei). Arrows: prominent NMMIIA aggregates (Scale bar: 100 μm).

To explore whether podocytes show a different phenotype compared with fibroblasts, we transfected immortalized podocytes as well as primary WT fibroblasts with vectors carrying V5-tagged NMMIIA WT or p.(Arg424Gly) coding sequences. Podocytes transfected with the Arg424Gly sequence showed prominent NMMIIA aggregates compared with transfection with the WT sequence (Figure 4a). In contrast, in transfected fibroblasts only small punctate structures were observed that differ markedly in size and morphology from podocyte aggregates (Figure 4b), and WT-like filamentous NMMIIA distribution was maintained, consistent with our observations in patient-derived fibroblasts (Figure 3a). These results demonstrate that the NMMIIA aggregates observed in podocytes are not an artefact of overexpression and fibroblasts are not suitable to test the effect of the p.(Arg424Gly) NMMIIA variant. However, suitability could be variant-dependent.

### Patient-Derived Blood Cells Exhibit Cell Type Specific Mechanical and Morphological Changes

**Figure 5:**
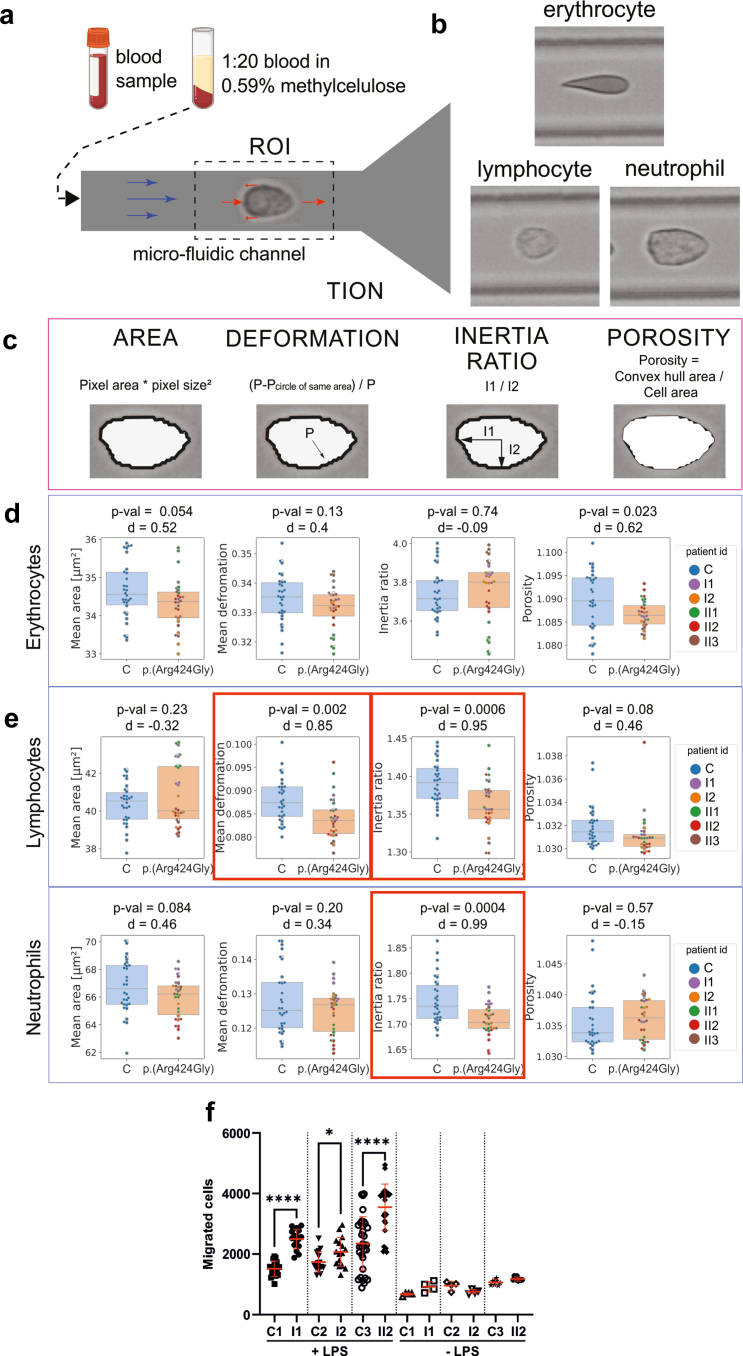
Effect of p.(Arg424Gly) on blood cell features. (a) Blood samples are diluted in methylcellulose and measured in the region of interest inside a micro-fluidic channel where hydrodynamic net forces induce cell deformation at high throughput (∼1,000 cells/sec). (b) Representative images of analyzed cell types. (c) Schematic representation of measured cell shape features. (d) Cell features of patient vs control erythrocytes. (e) Cell features of patient vs control leukocytes. Data is shown as box plots with single values adjusted to be nonoverlapping for 30 control individuals and 5 p.(Arg424Gly) patients, each measured 6 times. Red boxes mark cell features that were significantly different after Holm-Bonferroni correction. P-values (t-test) and effect sizes are reported. (f) Monocytes were isolated from patient blood and used in a 5 μm Boyden chamber migration assay with 100 ng/ml lipopolysaccharide as a stimulant. Cell counts were measured fluorometrically. Graph represents mean ± SD. Mann-Whitney tests were used to compare each patient sample with an age- and sex-matched control. ∗*P* ≤ 0.05, ∗∗∗∗*P* ≤ 0.0001.

Given that the elevated motor activity of the p.(Arg424Gly) variant may influence cell deformability, and *MYH9* pathogenic variants are known to cause prominent blood cell phenotypes, we conducted deformability cytometry^24^ on patient-derived blood cells (Figure 5a). This technique enables the analysis of the mechanical and morphological properties of blood cells (Figure 5b).^40^ The core principle involves flowing cells through a narrow microfluidic channel, where they deform on a millisecond timescale under controlled shear stress. Several studies addressed the effect of NMMIIA knockdown on cellular deformability using deformability cytometry, showing both decreased^41^ and increased^42^ deformation with myosin IIA inhibition. We compared the morphological characteristics of blood cells from 5 patients with 30 controls, focusing on the following 4 shape features: cell area, deformation, inertia ratio, and porosity (Fig. 5C). Cell inertia ratio defined as I= √(I1/I2) where I1 and I2 are 2 principal components of the moment of inertia. Inertia ratio can also be interpreted as the ratio of a long over short axes of the best fitting ellipse to the cell shape. Cell porosity is a dimensionless ratio calculated as the area of the convex hull divided by the original cell area, and it quantifies the degree of enclosure of the cell shape. Morphological analysis revealed no statistically significant differences in erythrocytes (Figure 5d). However, white blood cells exhibited several morphological changes (Figure 5e). Specifically, patient lymphocytes were less deformed (0.084 ± 0.004) compared with controls (0.088 ± 0.005), and their inertia ratio was lower (1.36 ± 0.03 vs. 1.39 ± 0.03). A similar trend was observed in neutrophils, where the inertia ratio in patient cells was 1.71 ± 0.03 compared with 1.75 ± 0.05 in controls, indicating a more rounded cellular shape under shear stress. The more roundly shaped lymphocytes and neutrophils which express the p.(Arg424Gly) variant heterozygously suggest a stiffening of these cells during short timescales.

### Patient-Derived Monocytes Exhibit Enhanced Migratory Capacity in Response to Lipopolysaccharide

Monocytes depend on NMMII for motility and differentiation to macrophages,^43^^,^^44^ but were absent in relevant numbers in our cytometry measurements. We, therefore, enriched monocytes from 3 affected family members (I1, I2, II2), and performed Boyden chamber migration assays^45^ with lipopolysaccharide as a stimulant. All patient monocytes migrated faster than the controls (Figure 5f).

In summary, we identified a novel *MYH9* variant within the highly conserved motor domain O-helix that causes *MYH9*-RD without Döhle-like bodies and NMMIIA-aggregates in blood cells. The p. Arg424Gly variant leads to an accelerated actin-activated ATP turnover and an increased motor activity, which suggests that it is a gain-of-function mutation.

## Discussion

Following the initial presentation of the index patient I1 with chronic kidney disease of unknown origin, the clinical data from him and 4 additional affected family members are consistent with the diagnosis of *MYH9*-RD. However, the absence of macrothrombocytopenia and Döhle-like bodies in granulocytes diverges from the traditionally defined phenotype of *MYH9*-RD. The phenotype varies within the family, but consistently involves multiorgan progression into a syndromic pattern. Elevated liver enzymes without impaired liver function are a frequent feature^46^ that may be caused by NMMIIA related altered bile canicular contraction^47^^,^^48^ or modified trafficking of bile salts as shown in Madin-Darby canine kidney cells.^49^

*MYH9*-RD has been associated with Alport syndrome based on focal thickening and thinning or splitting of the basal membrane,^50^^,^^51^ but it also showed foot process effacement, mesangial cell proliferation, and matrix expansion on renal histopathology,^52^ which matches the kidney biopsy findings of our patients (Table 1).

Similar renal involvement has been reported for NMMIIA variants that also presented with giant platelets and in some cases with intermittently normal platelet counts.^53^ Furthermore, variable expressivity of symptoms, as well as the apparent differences in diagnoses between unrelated and related patients have been described before,^54^ making it apparent that this novel variant belongs to the spectrum of *MYH9*-RD despite lacking common diagnostic markers.

The p. Arg424Gly variant is located in the NMMIIA motor domain O-helix, the latter of which is highly conserved across all human myosin isoforms and throughout the species. This highlights the crucial role of this arginine residue for NMMIIA function. Another pathogenic variant at position 424 of NMMIIA (p.Arg424Gln) was previously reported^55^ in a 25-year-old female patient with chronic kidney disease of unknown origin and a family history of kidney disease. No functional testing was carried out. The same variant was reported recently in an 18-year-old female patient and her 53-year-old father, both with proteinuria beginning in early childhood and renal disease as the predominant disorder.^56^ Both showed an incomplete Epstein–Fechtner syndrome with mild thrombocytopenia and slightly elevated transaminases comparable to our patients. No bleedings were reported. These findings underline the importance of this specific arginine in the maintenance of renal function.

The precise mechanism by which the variant p. (Arg424Gly) contributes to the proteinuric phenotype remains to be fully elucidated. However, our experiments show that the pathogenic variant increases the ATP turnover rate (*k*_cat_) of NMMIIA. Single turnover experiments with fluorescent *mant*-ATP support these findings, showing increased rates of ATP binding and ADP release for the p.(Arg424Gly) protein. Importantly, the changes observed for the enzymatic activity of the motor also translate into enhanced motor function resulting in faster translocation of actin filaments both in the absence and presence of an external load. The effect of mutations in the motor domain on ATP turnover has only been investigated for 2 other mutations (p.Arg702Cys, p.Asn93Lys) before and both are reported to decrease ATP turnover.^57^ We have also determined the effect of the known pathogenic variants p.Arg702Cys^33^^,^^34^ and p.Arg1162Ser^35^ on NMMIIA motor activity. We measured reduced sliding velocity of varying degree for all 3 variants, which validates the previous results for p.Arg702Cys^57^ and provides new information on the molecular mechanisms of p.Arg705His and p.Arg1162Ser (Supplementary Figure S3).

Other studies have mainly focused on mutations in the coiled-coil tail domain and their effect on filament formation.^58^^,^^59^ To date, no mutation has been reported to increase ATP turnover and motor function (gain-of-function), as we observed for the p.(Arg424Gly) variant.

Dysregulation of the actin cytoskeleton remains the most common factor contributing to foot process effacement and proteinuria.^60^ We suggest that the presented enhanced intrinsic motor activity of the p. (Arg424Gly) variant is a central contributor to the disease mechanism. This might impact the fine-tuning of actin cytoskeletal processes that cause the kidney phenotype of our patients, though the available data do not allow direct inference about the behavior of mixed WT/mutant NMMIIA filaments, since the used HMM constructs lack the physical interactions required to model filament assembly and dominance.

Fibroblasts have been used to study pathogenic variants with a predominant podocyte phenotype before.^36^^,^^37^ However, functional testing in patient-derived primary fibroblasts showed no impairment regarding NMMIIA-driven migration^61^ and autophagy^62^ as well as no NMMIIA aggregates or changes in focal adhesion density. Only changes in cell shape could be observed, which has also been shown for podocytes with a p.(Glu1841Lys) *MYH9* variant.^63^

To directly compare the consequences of p.(Arg424Gly) in podocytes and fibroblasts, we overexpressed p.(Arg424Gly) in immortalized human podocytes and primary control fibroblasts, which led to the formation of NMMIIA aggregates in podocytes, whereas fibroblasts showed no relevant aggregates. This indicates increased sensitivity of podocytes to the variant.

The p.(Arg424Gly) variant does not induce Döhle-like bodies or NMMIIA aggregates in granulocytes, despite the high expression of NMMIIA in various blood cell types.^64^^,^^65^ To investigate potential subtle alterations in the actin cytoskeleton of these cells, we employed deformability cytometry. The analysis revealed significant changes in the deformability of patient-derived lymphocytes, in which NMMIIA plays a role in immunological synapse maturation.^66^^,^^67^ Monocytes are amoeboid-like fast migrating cells and depend on NMMII for their motility and differentiation to macrophages.^43^^,^^44^ NMMIIA is primarily responsible for cell migration by facilitating the generation of contractile forces, whereas NMMIIB contributes to maintaining cellular tension and stability.^61^^,^^68^ In line with the observed enhanced motor function of the p. Arg424Gly variant, we observed an increased monocyte migration speed in 3 of our patients compared with controls. Biallelic expression of NMMIIA would rather suggest stalling by formation of NMMIIA bundles of normal and altered proteins. However, monoallelic expression of NMMIIA has been suggested for bone marrow mononuclear cells, including lymphocytes, neutrophils, and monocytes (see Supplementary Table S2 of^69^), underlining the suggested cell-specific effects of this variant. Interestingly, the patients do not experience frequent or severe infections, nor do they show signs of autoimmune diseases. This observation suggests that functional effects of this variant may be present in multiple cell types, but not uniformly pathological across all affected cell types.

In conclusion, we identified a novel *MYH9* variant located within the highly conserved motor domain O-helix causing *MYH9*-RD without the typical Döhle-like bodies or NMMIIA aggregates. Through a range of cell biological, biochemical, and biophysical assays, we traced its pathogenicity from the molecular to the cellular level, shedding light on the cell-specific mechanisms underlying *MYH9*-RD. Based on these findings, we report the first gain-of-function variant of *MYH9*. We propose that the enhanced intrinsic motor activity of the p.(Arg424Gly) variant represents a central aspect of the disease mechanism, whereas impaired actomyosin dynamics, resulting from the incorporation of the p.(Arg424Gly) variant into NMMIIA filaments and higher-order actomyosin assemblies, remains speculative. Furthermore, our findings underline the clinical complexity of *MYH9*-RD.

## Disclosure

None of the authors have competing financial interests to disclose.

## Patient Consent

This study was approved by the Ethics Committee of the Friedrich-Alexander University Erlangen-Nuremberg, Germany (protocol no. 251_18 B, 549_20 B, 295_20B and 22-150-D). It adheres to the Declaration of Helsinki. Informed consent was obtained from all participants.

## Supplementary Material

Supplementary File (PDF) **Supplementary Materials and Methods**. **Supplementary References. Figure S1.** DNA sequence chromatograms of all affected family members showing the heterozygous variant c.1270C>G in MYH9. **Figure S2.** Multiple-sequence alignment of the sequence region in the O-helix surrounding the mutated residue Arg424 of multiple nonmuscle myosin isoforms. **Figure S3.** Effect of mutations p.Arg702Cys, p.Arg705His and p.Arg1162Ser on unloaded NMMIIA motor activity. **Figure S4.** Patient derived fibroblasts do not show differences in migratory behavior.

## Footnotes

Supplementary File (PDF)

**Supplementary Materials and Methods**

**Supplementary References**
**Figure S1.** DNA sequence chromatograms of all affected family members showing the heterozygous variant c.1270C>G in MYH9. **Figure S2.** Multiple-sequence alignment of the sequence region in the O-helix surrounding the mutated residue Arg424 of multiple nonmuscle myosin isoforms. **Figure S3.** Effect of mutations p.Arg702Cys, p.Arg705His and p.Arg1162Ser on unloaded NMMIIA motor activity **Figure S4.** Patient derived fibroblasts do not show differences in migratory behavior. STROBE checklist
