# Supplementary material for: MYH9 Variant p.(Arg424Gly) Alters Nonmuscle Myosin IIA Contraction, Causing Atypical MYH9-related Disease
Source: Kidney Int Rep. 2026 Feb 3;11(4):106343. doi: 10.1016/j.ekir.2026.106343 (PMC12966671; doi:10.1016/j.ekir.2026.106343)
Supplement: Supplementary File (PDF) — Supplementary Materials and Methods. Supplementary References. Figure S1. DNA sequence chromatograms of all affected family members showing the heterozygous variant c.1270C>G in MYH9. Figure S2. Multiple-sequence alignment of the sequence region in the O-helix surrounding the mutated residue Arg424 of multiple nonmuscle myosin isoforms. Figure S3. Effect of mutations p.Arg702Cys, p.Arg705His and p.Arg1162Ser on unloaded NMMIIA motor activity. Figure S4. Patient derived fibroblasts do not show differences in migratory behavior. [file mmc1.pdf]

Supplementary Material

- Supplementary Materials and Methods
- Supplementary Figure S1: DNA sequence chromatograms of all affected family members showing the heterozygous variant *c.1270C>G* in *MYH9*.Supplementary references
- Supplementary Figure S2: Multiple-sequence alignment of the sequence region in the O-helix surrounding the mutated residue Arg424 of multiple non-muscle myosin isoforms.
- Supplementary Figure S3: Effect of mutations p.Arg702Cys, p.Arg705His and p.Arg1162Ser on unloaded NMMIIA motor activity
- Supplementary Figure S4: Patient derived fibroblasts do not show differences in migratory behaviour.
- Supplementary references
- STROBE checklist

Supplementary Material and Methods

Patient and ethical approval

The study was approved by the Ethics Committee of the University Hospital, Erlangen, Germany (protocol no. 251_18 B and 549_20 B). The use of archived material was approved by the Ethics Committee of the Friedrich-Alexander University of Erlangen-Nuremberg, which waived the need for retrospective consent for the use of archived rest material (Re.-No. 22-150-D). The study adheres to the Declaration of Helsinki. Informed consent was obtained from all participants.

Clinical workup

Whole exome sequencing (WES)

WES of the index patient`s DNA (Illumina HiSeq 2500, Illumina, San Diego, CA, USA) and processing of the raw data (alignments and variants calls, subsequent filtering and inspection) was performed as described previously.[12] All relevant variants were confirmed, and then analyzed in the affected family members by Sanger sequencing. The variant was interpreted according to the standards and guidelines of the American College of Medical Genetics and Genomics (ACMG) [13] and the Combined Annotation Dependent Depletion (CADD) score[14].

PAS staining

Kidney biopsies were fixed in 4% formalin buffered in 0.1M PBS (pH 7.6), followed by serial dehydration and paraffin embedding. Afterwards, 2 µm thick sections were cut, deparaffinized, rehydrated, and stained with periodic acid–Schiff (PAS) according to pathology standard procedure.

Electron microscopy

Kidney biopsies were fixed in 4% formalin buffered in 0.1M PBS (pH 7.6). After that, a portion of the formalin-fixed biopsy containing glomeruli was washed in 0.1 M PBS (pH 7.6), treated with 0.5% OsO_4 for 60 minutes, and stained with 1% uranyl acetate in 70% ethanol. After dehydration, tissue blocks were embedded in epoxy araldite resin (Serva Electrophoresis GmbH, Heidelberg, Germany). Ultrathin 80 nm sections were cut on an UC6 ultramicrotome (Leica Microsystems; Daher Corporation, Washington D.C., USA), rinsed in lead citrate buffer before analysis, and analyzed on a transmission electron microscope (LEO 910, Carl Zeiss AG, Oberkochen, Germany) at 80 kV.

Blood smear staining

Drops of patient blood were prepared as blood smears on glass slides, dried, stained and imaged. Pappenheim's staining was performed according to May-Grünwald-Giemsa. Immunofluorescence (IF) stainings were performed using an anti-NMMIIA antibody (rabbit; 1 mg/ml; 1:200; Biolegend; Cat. No. 909801; PerkinElmer, Waltham, MA, USA) and Diamidin-2-phenylindol (DAPI; 1 mg/ml; 1:5000; Cat. No. 62248; Thermo Fisher Scientific, Waltham, MA, USA). Imaging was done using a Zeiss LSM800 confocal laser scanning microscope (Carl Zeiss AG, Oberkochen, Germany) with a 63x magnification objective on 3x zoom. Images were processed using ZEN blue software (Carl Zeiss AG, Oberkochen, Germany).

In vitro analysis of contraction velocity

Plasmids for recombinant protein production

The generation of the pFastBac1 vectors encoding the NMMIIA- heavy meromyosin-like (HMM) fragment (1-1337 aa) (Uniprot-ID: P35579) fused to an Avi-His_8-tag and the NMMIIA- two spectrin-like repeats (2R) motor domain construct (1-777 aa) carrying a C-terminal His_8-tag was previously described.[15] Vectors carrying NMMIIA-HMM p.(Arg424Gly) and NMMIIA-2R p.(Arg424Gly) were generated by site-directed mutagenesis using the WT vectors as templates. Successful site-directed mutagenesis was confirmed by Sanger sequencing performed by Microsynth Seqlab (Göttingen, Germany). The human essential light chain MLC6a (Uniprot-ID: P60660) and the human regulatory light chain MLC12b (Uniprot-ID: O14950) were cloned into the pFastBacDual vector for the purpose of co-production with NMMIIA-HMM in the *Spodoptera frugiperda* (Sf9) system. The coding sequence of human cytoskeletal β-actin (Uniprot-ID: P60709) was fused via a C-terminal linker to a His-tagged thymosin β4 moiety (Uniprot-ID: P62328). The fusion protein was cloned into the multiple cloning site of the pFastBac-Dual vector under control of the polyhedrin promotor. The recombinant bacmids and virus required for the production of the myosins and actin in the Sf9 system were produced according to the Bac-to-Bac baculovirus expression system protocol (Thermo Fisher Scientific, Waltham, MA, USA).

Protein Production and Purification

NMMIIA-HMM WT/p.(Arg424Gly), NMMIIA-2R WT/p.(Arg424Gly) and the β-actin-thymosin β4-His_6 fusion protein were produced in the *Spodoptera frugiperda* (Sf9) expression system. NMMIIA-HMM was co-produced with the human light chains MLC6a and MLC12b. Sf9 cells (2×10^6 cells mL^{-1}) were infected 1:50 with the

corresponding virus. Protein production was carried out over 72 hours. After 72 hours, the cells were pelleted, washed with PBS, shock frozen in liquid nitrogen and stored at -80 °C. For a typical preparation, cell pellets from two liters of Sf9 culture were used. NMMIIA-HMM WT and mutant proteins were purified using Ni-NTA affinity-chromatography followed by size exclusion chromatography, as previously described.[16,17] NMMIIA-2R WT and mutant proteins were purified as previously described for NMMIIC.[18] NMMIIA-HMM requires phosphorylation of the associated regulatory light chain (MLC12b) for full enzymatic activity. Therefore, purified NMMIIA-HMM WT and p.Arg424Gly was incubated with myosin light chain kinase (molar ratio: 10:1) in phosphorylation buffer (25 mM MOPS, pH 7.3, 50 mM KCl, 5 mM $MgCl_2$, 1 mM $CaCl_2$, 0.2 µM calmodulin, 3 µM regulatory light chain (MLC12b), 3 µM essential light chain (MLC6a), 1 mM DTT and 1 mM ATP) for 30 minutes at 30 °C before being used in assays.

Recombinant human β-actin was purified was purified as previously described for human γ-actin.[15] α-skeletal actin was purified from chicken pectoralis major muscle, as previously described for rabbit α-skeletal actin.[20]

Steady-state ATPase assay

The basal and actin-activated steady-state ATPase of NMMIIA-HMM WT and p.(Arg424Gly) was measured using a NADH-coupled enzymatic assay. Experiments were performed with 0.5 µM HMM and increasing concentrations of α-skeletal actin at 30 °C in assay buffer (25 mM HEPES pH 7.3, 5 mM $MgCl_2$, 50 mM KCl, 0.5 mM DTT). The assay buffer is supplemented with the enzymatic system, which generates the quantifiable readout (1 mM ATP, 0.5 mM NADH, 0.5 mM 2-phosphoenolpyruvate, 0.02 mg mL^{-1} lactate dehydrogenase, 0.05 mg mL^{-1} pyruvate kinase, 0.5 mM NADH). The change in absorbance at 340 nm due to oxidation of NADH was recorded in a Multiskan FC Microplate Photometer (Thermo Fisher Scientific, Waltham, MA, USA). The ATP turnover of myosin at the various actin concentrations was determined by fitting linear equations to the primary data. The change of ATP turnover over increasing actin concentrations is best described by the Michaelis-Menten equation, which yields k_{cat} (maximum value of ATP turnover at saturating actin) and K_{app} (actin concentration at half-maximal activation of ATP turnover). k_{basal} indicates the ATP turnover rate in the absence of actin.

Unloaded and loaded in vitro motility assay

The productive interaction between NMMIIA-HMM WT or p.(Arg424Gly) and F-actin was analyzed in the *in vitro* motility assay.[21] We used HMM fragments of NMMIIA WT and p.(Arg424Gly) that form hexamers consisting of two C-terminally truncated heavy myosin chains and two essential and two regulatory light chains, as they are ideal for studying myosin motor function. For the use in the *in vitro* motility assay, actin filaments were labelled overnight with a 1.5-fold excess of phalloidin-tetramethylrhodamine B-isothiocyanate (Merck KGaA, Darmstadt, Germany). The *in vitro* motility experiment was performed as described in the following. Flow cells for use in the in vitro motility assay were constructed using nitrocellulose-coated cover slips. The solution containing NMMIIA-HMM was flushed into the flow cell and incubated for three minutes. The flow cell was then washed and blocked with BSA-buffer (25 mM imidazole pH 7.4, 50 mM KCl, 5 mM $MgCl_2$, 1 mM EGTA, 10 mM DTT, 0.5 mg mL^{-1} BSA). The inactive myosin heads were then blocked with 2 µM unlabelled WT actin. The following ATP-wash removed unlabelled actin only from intact myosin heads, thus the inactive myosin heads do not interfere with the measurement. Remaining free ATP was removed by flushing BSA-buffer into the flow cell. A solution containing 30 nM labelled actin filaments was applied to the flow cell and incubated for three minutes. After three minutes the assay was started by applying assay buffer (25 mM Imidazole pH 7.4, 50 mM KCl, 5 mM $MgCl_2$, 1 mM EGTA, 0.5% methyl cellulose (15 cP, Sigma), 10 mM DTT, 4 mM ATP, 5 mg mL^{-1} glucose, 0.1 mg mL^{-1} glucose oxidase, 0.02 mg mL^{-1} catalase) to the flow cell. The flow cell was sealed with Korasilon®-paste (Obermeier, Bad-Berleburg, Germany) to prevent evaporation of the sample. Unloaded *in vitro* motility experiments were performed at constant 37 °C using an Olympus IX70 inverted fluorescence microscope (Olympus, Hamburg, Germany) equipped with a 60×/1.49 NA PlanApo oil immersion objective and an Orca Flash 4.0 CMOS camera (Hamamatsu Photonics Deutschland GmbH, Herrsching, Germany). For the loaded *in vitro* motility assay, slight modifications of the protocol were necessary. Inactive myosin heads were removed by high-speed centrifugation (136,000 × g) in the presence of F-actin and ATP before surface-immobilization. Human α-actinin was immobilized on the nitrocellulose-coated surface together with NMMIIA-HMM to generate a viscoelastic load on the actin filaments. Experiments were performed at 21 °C to minimize the observed rupture of the filaments at high α-actinin concentrations.

Analysis of the recorded image series was performed in ImageJ using the plugin wrMTrck.[22] The plugin was used to determine the trajectories and corresponding velocities of the individual actin filaments. Only filaments that showed movement for at least 30 consecutive seconds were tracked and used to determine the average sliding velocity. The average sliding velocity was determined from the Gaussian distribution obtained from the velocities of the individual filaments.

Data obtained from loaded in vitro motility experiments were fitted using the following dose-response function:

$$y = v_{min} + \frac{(v_{max} - v_{min}) \cdot x^n}{IC_{50}^n + x^n}$$

Here, v_{min} and v_{max} represent the minimal and maximal observed sliding velocities, respectively. IC_{50} denotes the concentration of α-actinin that results in half-maximal inhibition, and *n* is the Hill coefficient, which determines the steepness of the dose-response curve.

Myosin single-turnover experiments

The effect of p.(Arg424Gly) on ATP binding, hydrolysis, and ADP release was investigated by performing single-turnover experiments with the fluorescent ATP-analogue *mant*-ATP (2′-/3′-O-(N′-methylanthraniloyl)-ATP) (Jena Bioscience, Jena, Germany). We used constitutively active, single-headed NMMIIA-2R constructs generated by fusing the NMMIIA motor domain with a rigid, artificial lever arm consisting of two spectrin-like repeats[32]. Actomyosin and *mant*-ATP were rapidly mixed in a 1:1 ratio to a final concentration of 0.25 µM NMMIIA-2R WT/p.(Arg424Gly), 10 µM α-skeletal actin and 0.1 µM mantATP in assay buffer (20 mM MOPS pH 7.0, 100 mM KCl, 5 mM $MgCl_2$) using a HiTech Scientific SF61 stopped-flow system (TgK Scientific Limited, Bradford on Avon, UK) at a constant 21°C. *mant*-ATP fluorescence was excited at 365 nm and monitored through a KV389 cut-off filter. The recorded traces are best described by a double-exponential function, which yields the rate of mant-ATP binding ($k_{ATP\text{-}on}$) and the rate of mant-ADP release ($k_{ADP\text{-}off}$). To generate a quantifiable metric reflecting overall ATP turnover and its alteration upon mutation, we measured the time delay between the midpoint of the fluorescence rise and the midpoint of the fluorescence decay in both WT and mutant experiments (full width at half maximum (FWHM) of the experimental traces).

Statistical analysis and visualization

Statistical analysis and visualization were performed using GraphPad Prism 7 (GraphPad Software LLC., Boston, MA, USA) and Origin 2023 (OriginLab Corporation. Massachusetts, USA). Comparison between groups was performed using Kruskal-Wallis tests or two-sample t-test. p values were defined as followed: * = $p < 0.05$; ** = $p < 0.01$; *** = $p < 0.001$. Assessment and visualization of the potential implications of variant p.(Arg424Gly) for the NMMIIA structure was performed using ChimeraX. [12]

Cell culture

Generation of primary fibroblasts

Human dermal fibroblasts were generated from sterile skin punches cultured in Dulbecco's Modified Eagle's Medium (DMEM; Gibco, Thermo Fisher Scientific, Waltham, MA, USA) supplemented with 10% fetal calf serum (FCS), 1% sodium pyruvate and 1% penicillin and streptomycin (P/S) in 5% CO2 at 37°C as described before.[S1] Control fibroblasts were obtained from a healthy volunteer (sex-matched). Fibroblasts were maintained in DMEM supplemented with 10% FCS and 1% P/S. Fibroblasts from passages 5-9 were used for the experiments.

Cell size measurement

5000 primary fibroblasts were grown to full density on glass coverslips, fixed in 4% PFA in PBS, and were immunofluorescently stained using an anti-NMMIIA antibody (rabbit; 1 mg/mL; 1:200; Biolegend; Cat. No. 909801; PerkinElmer, Waltham, MA, USA) and Hoechst 33258 (10 mg/mL; 1:2000; Cat. No. B2883; Merck, Darmstadt, Germany). Imaging was done using a Leica DM6000B fluorescent microscope (Leica Microsystems; Daher Corporation, Washington D.C., USA) on 20x magnification. Image analysis was performed using ImageJ Fiji software (ImageJ 1.53t). Cell size measurement was done by hand using the polygon selection tool. Partially visible and overlaying cells were excluded. 50 cells per type were analyzed.

Focal adhesion measurement

5000 primary fibroblasts were grown to full density on glass coverslips, fixed in 4% PFA in PBS, and were immunofluorescently stained using an anti-NMMIIA antibody (rabbit; 1 mg/mL; 1:200; Biolegend; Cat. No. 909801; PerkinElmer, Waltham, MA, USA), an anti-Vinculin antibody (mouse; 1:200; Novus Biological; Cat. No. NB600-1293; Bio-Techne, Minneapolis, MN, USA) and Hoechst 33258 (10 mg/mL; 1:2000; Cat. No. B2883; Merck, Darmstadt, Germany). Imaging was done using a Leica

DM6000B fluorescent microscope (Leica Microsystems; Daher Corporation, Washington D.C., USA) on 20x magnification. Image analysis was performed using ImageJ Fiji software (ImageJ 1.53t). Individual cell area measurements were performed by hand using the Polygon selection tool. Partially visible and overlaying cells were excluded. Focal adhesions of individual cells were measured using the Analyze Particles tool. Focal adhesions were then normalized to the number of cells or to 1 μm^2 of cell area. 10 cells per type were analyzed.

Autophagy assay

50,000 primary fibroblasts were seeded in 6 well plates and grown to full density. At confluence, the cells were either starved for 24 hours or kept in growth medium, before 50 µM bafilomycin A1 (BafA; Cat. No. SML1661; Merck, Darmstadt, Germany) or DMSO treatment took place for 24 hours. Afterwards, protein was extracted from the cells and Western Blotting was performed with 25 µg protein lysate. Blots were stained using an anti-LC3b antibody (rabbit; 1.0 mg/mL; 1:1500; Novus Biological; Cat. No. NB100-2220; Bio-Techne, Minneapolis, MN, USA). Loading control was achieved by total protein staining (Ponceau S solution; Cat. No. P7170; Merck, Darmstadt, Germany). LC3b I and LC3b II were normalized to total protein. The ratio between normalized LC3b II and LC3b I was measured densitometrically using ImageJ Fiji software (ImageJ 1.53t).

Wound healing assay using scratching

5000 primary fibroblasts were seeded in 24 well plates on glass coverslips and grown to full density. Once at confluence, the cell layer was gently scraped in a straight line in the middle of each well with a 1 mm pipette tip. After that, the cell monolayer was washed gently with Dulbecco’s phosphate buffered saline (DPBS; Gibco; Thermo Fisher Scientific, Waltham, MA, USA) and replenished in fresh low serum medium supplemented with 5% FCS and 1% P/S. Imaging took place at 0 and 12 hours post scratching using an EVOS M5000 Microscope (Thermo Fisher Scientific, Waltham, MA, USA) on 10x magnification. Image analysis was performed using ImageJ Fiji software (ImageJ 1.53t).

Wound healing assay using inserts

5000 primary fibroblasts were seeded in 4 well plates on glass coverslips inside 2 well wound healing inserts (Cat. No. 81176; ibidi GmbH, Gräfelfing, Germany) and grown to full density. At confluence, the inserts were removed gently. The monolayer was

washed with DPBS and replenished in fresh low serum medium supplemented with 5% FCS and 1% P/S. Imaging took place at 0 and 12 hours post insert removal using an EVOS M5000 Microscope (Thermo Fisher Scientific, Waltham, MA, USA) on 10x magnification. Image analysis was performed using ImageJ Fiji software (ImageJ 1.53t).

Plasmids for transfection

The plasmid pMYH9-WT-V5 (Plasmid #183512; Addgene, Watertown, MA, USA) was used as a template for site-directed mutagenesis (*c.1270C>G*, p.(Arg424Gly)) using 5-GGCGACCTATGAGGGGATGTTCCGCTGGCT -3 and 5-TTGGCCAAGGCCTCGATGGCAAAGTCAGCC -3 as primers. Success of mutagenesis was confirmed by Whole Plasmid Next Generation Sequencing (Genewiz, Azenta, Burlington, MA, USA).

Transfection of fibroblasts

25,000 primary fibroblasts were grown to 80% density on glass cover slides and transfected with either the WT plasmid or the one containing the mutated NMMIIA using Lipofectamine 3000 (Thermo Fisher Scientific, Waltham, MA, USA) according to the manufactorer's manual. After 48 hours, the cells were fixed in 4% PFA in PBS and stained immunofluorescently using an anti-NMMIIA antibody (rabbit; 1 mg/mL; 1:250; Biolegend; Cat. No. 909801; PerkinElmer, Waltham, MA, USA), an anti-V5 (mouse; 1:200; Cell Signaling; Cat. No. 80076S; Danvers, MA, USA), and Hoechst 33258 (10 mg/mL; 1:2000; Cat. No. B2883; Merck, Darmstadt, Germany). Imaging was done using a Leica DM6000B fluorescent microscope (Leica Microsystems; Daher Corporation, Washington D.C., USA) on 20x magnification.

Transfection of podocytes

180,000 immortalized human podocytes were seeded in cell culture flasks and differentiated for ten days on 37°C. They were transfected using an electroporation chamber (270V) and put on glass cover slides. After 48 hours, the cells were fixed in 4% PFA in PBS and stained immunofluorescently using an anti-NMMIIA antibody (rabbit; 1 mg/mL; 1:250; Biolegend; Cat. No. 909801; PerkinElmer, Waltham, MA, USA), an anti-V5 (mouse; 1:200; Cell Signaling; Cat. No. 80076S; Danvers, MA, USA), andHoechst 33258 (10 mg/mL; 1:2000; Cat. No. B2883; Merck, Darmstadt, Germany). Imaging was done using a Leica DM6000B fluorescent microscope (Leica Microsystems; Daher Corporation, Washington D.C., USA) on 20x magnification.

Blood cells

Deformability cytometry (DC)

DC measurements of 10 µL whole blood were performed as described previously using an AcCellerator instrument (Zellmechanik Dresden, Dresden, Germany)[24,S2]. The following changes have been made in our protocol. An aliquot of 50 µL of a mixture of 10 µL of whole blood with 190 µL of a measurement buffer (0.6% w/v Methylcellulose in PBS, adjusted to a viscosity of 25 mPa/s) was reversely taken up into the tubing provided with the AcCellerator facilitating the syringe pump at -2 µL/s flow rate. This tubing was connected to the sample inlet of a prefilled (measurement buffer through the sheath inlet) PDMS-based microfluidic chip containing a 20 x 20 µm and 500 µm long channel constriction. The cells were flown through this channel at a total flow rate of 0.06 µL/s (sample flow 0.015 µL/s, sheath flow 0.045 µL/s). By this flow the cells get deformed by hydrodynamic stresses as they pass through the channel. The cells get imaged at a frame rate of 3400 Hz at the end of that channel using a ROI size of 80 x 250 px. Images are segmented using custom trained machine learning segmentation algorithm based on small U-Net network [S3]. Cell features are calculated from binarized images using dcnum software (https://github.com/DC-analysis/dcnum). Upon feature extraction, selection of different blood cell types was done using an algorithm based on box filters and unsupervised clustering of cell features [S4]. Maximal porosity of the analyzed white blood cells (WBC) was set to 10% instead of 5% as in previous protocol [13] to ensure the inclusion of all WBC.

Definition of cell features

Cell area is calculated as the first image moment of the cell contour. Cell deformation defined as: $1 - \frac{2\sqrt{\pi * A}}{P}$, where A denotes cell area and P cell perimeter. The second term in deformation formula is called circularity and per definition it equals one for a perfect circle. In this study, deformation is calculated from the cell contour rather than the convex hull, as used in previous studies[24]. Cell inertia ratio defined as $I = \sqrt{\frac{I_1}{I_2}}$ where I_1 and I_2 are two principal components of the moments of inertia. Inertia ratio can also be interpreted as ratio of a long over short axes of the best fitting ellipse to the cell shape. Cell porosity is a dimensionless ratio calculated as the area of the convex hull divided by the original cell area, and it quantifies the degree of enclosure of the cell shape.

Data analysis

In our two groups we have thirty control patients (mean age 35 ± 15, 63% females) and five patients with p.(Arg424Gly) mutation. Variance in each these two groups arises from error variance (between measurements) as well as from variance between subjects. To account for error variance arising from repeated measurement variability which is crucial in case of the small sample size, we have measured each p.(Arg424Gly) mutation patient six times. We do group comparison per measurement, having 30 control measurements and 30 p.(Arg424Gly) mutation group measurements (5x6). Comparison between groups is done with Student´s t-test, and effect size is calculated as Cohen´s d value. To account for multiple hypothesis testing (12 hypothesis) we corrected significance level with the Holm–Bonferroni correction.

Monocyte migration assay

Patient monocytes were isolated from blood samples using ROTI Sep (Carl Roth, Karlsruhe, Germany) and CD14-coupled microbeads (Miltenyi Biotec, Berisch Gladbach, Germany)[S5]. For each patient, monocytes from age and sex-matched control subjects were generated. After isolation, the monocytes were processed immediately using a Boyden chamber chemotaxis assay (96 wells, 5 µm pore size; Cat. No. ECM512; Merck, Darmstadt, Germany) according to the manufactoter's manual with 100 ng/ml LPS (Cat. No. L2880; Merck, Darmstadt, Germany) as a stimulant in the lower chamber. As migration medium we used RPMI-1640 supplemented with 1% FCS. After 4 hours of migration, cell counts were measured fluorimetrically by a Glomax Multi Detection System (Promega, Madison, WI, USA).

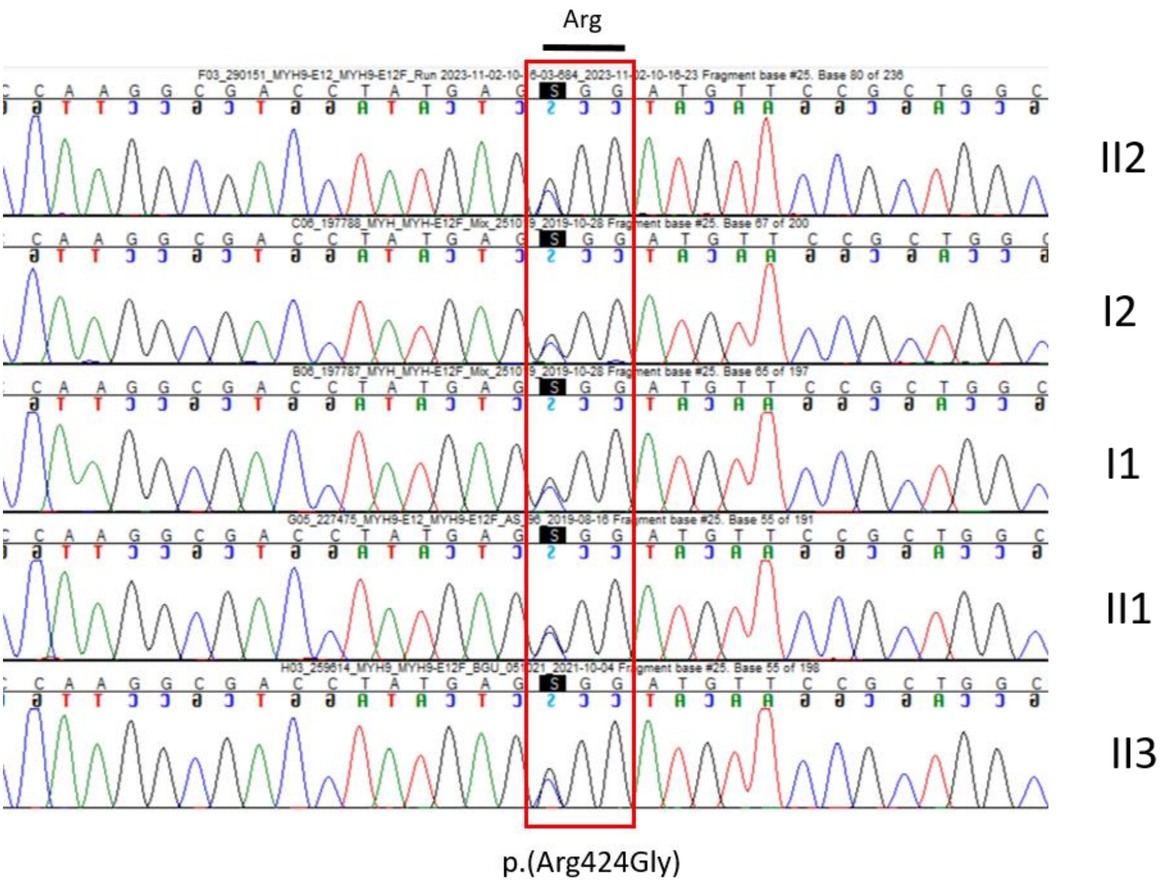

Supplementary Figure S1: DNA sequence chromatograms of all affected family members showing the heterozygous variant *c.1270C>G* in *MYH9*. Software used for sequence analysis: Sequencer v.5.4.6, GenCode, USA. MYH9 Ref. NM_002473.3.

	420				424				428
NM2A	A	T	Y	E	R	M	F	R	W
NM2B	A	T	Y	E	R	L	F	R	W
NM2C	A	T	Y	E	R	L	F	R	W
Myo1A	N	I	Y	S	R	L	F	D	W
Myo1E	A	L	H	A	R	V	F	D	F
Myo1H	A	V	Y	G	R	T	F	T	W
Myo3A	T	L	Y	G	R	L	F	S	W
Myo3B	A	L	Y	G	R	L	F	S	W
Myo5A	H	I	Y	A	K	L	F	N	W
Myo5B	H	I	Y	A	Q	L	F	G	W
Myo5C	K	I	Y	A	H	L	F	G	W
Myo6	T	V	Y	S	H	L	F	D	H
Myo7A	G	I	Y	G	R	L	F	V	W
Myo7B	G	I	Y	G	H	L	F	L	W
Myo9A	S	L	Y	S	A	L	F	D	W
Myo9B	S	L	Y	S	A	L	F	D	W
Myo10	A	L	Y	A	A	L	F	D	W
Myo15	V	L	Y	A	L	L	F	S	W

Supplementary Figure S2: Multiple-sequence alignment of the sequence region in the O-helix surrounding the mutated residue Arg424 of multiple non-muscle myosin isoforms. The alignment, which was carried out with all 27 human non-muscular myosin isoforms, shows a representative selection of sequences. The residue numbering corresponds to the NMMIIA sequence. The mutated residue in NMMIIA and the corresponding residues in the other non-muscle myosin isoforms are marked by the red box.

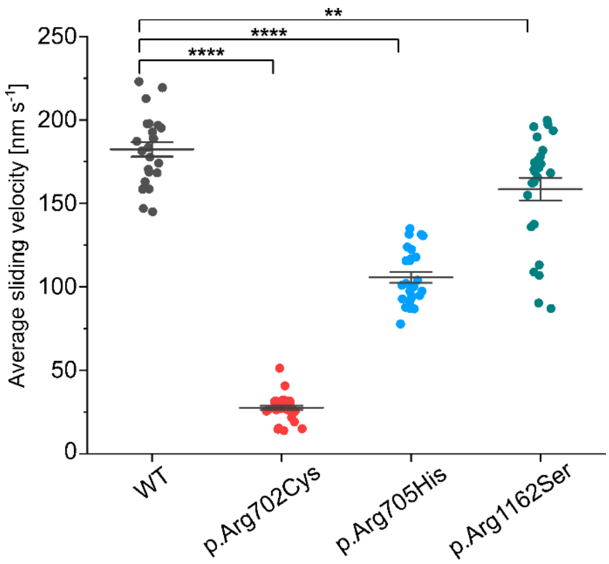

Supplementary Figure S3: Effect of mutations p.Arg702Cys, p.Arg705His and p.Arg1162Ser on unloaded NMMIIA motor activity. The effect of the mutations on NMMIIA motor activity was determined using the unloaded *in vitro* motility assay, as seen in Fig. 2. The average sliding velocity in each experiment was determined as described in Fig. 2. Data is shown as the mean ± SEM.

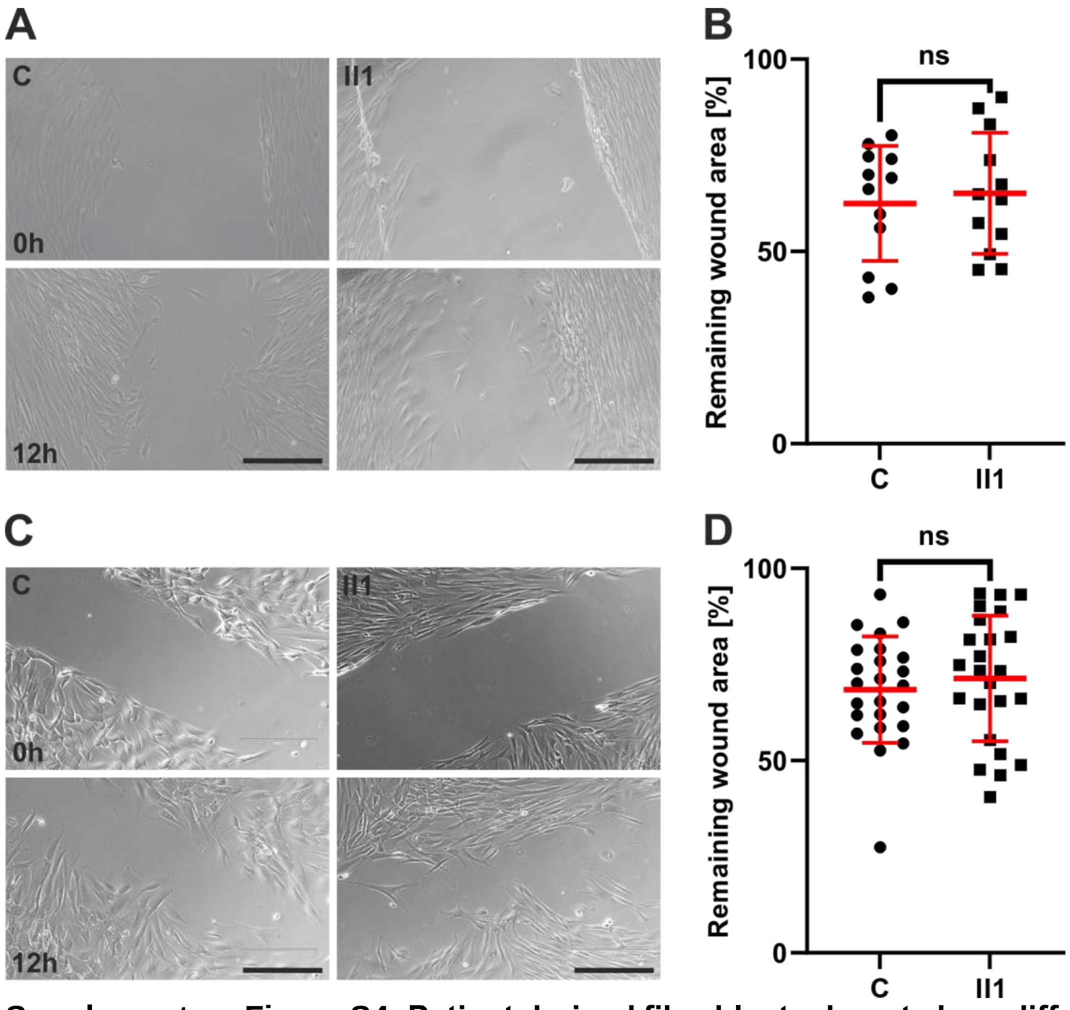

Supplementary Figure S4: Patient derived fibroblasts do not show differences in migratory behaviour. Fibroblasts were cultivated on glass cover slips with or without an ibidi barrier insert. **(A)** Images of fibroblasts at 0 h and 12 h timepoints post scratching with a 200 µl pipet tip. Scale bar: 100 µm. **(B)** Remaining wound area in percent. Mann-Whitney test was used to compare control and patient fibroblasts. **(C)** Images of fibroblasts at 0 h and 12 h timepoints post barrier removal (Scale bar: 100 µm). **(D)** Remaining wound area in percent. Kruskal-Wallis test was used to compare means of all groups. Images were analyzed using ImageJ.

Supplementary References

S1. Tiosano, D. *et al.* Mutations in PIK3C2A cause syndromic short stature, skeletal abnormalities, and cataracts associated with ciliary dysfunction. *PLoS Genet* **15**, e1008088 (2019).

S2 Toepfner, N. et al. Detection of human disease conditions by single-cell morpho-rheological phenotyping of blood. Elife 7(2018).

S3 Kaliman, S. et al. Small U-Net for Fast and Reliable Segmentation in Imaging Flow Cytometry. Cytometry Part A 107(2025).

S4 Kaliman, S. et al. Automation and improvement of WBC mechanical profiling in deformability cytometry. Biophysical journal (2025)

S5. Haberkamp, C. *et al.* TNF promotes DECTIN2 family C-type lectin receptor expression in human macrophages. *Journal of Leukocyte Biology* **113**, 615-625 (2023).

STROBE Statement—checklist of items that should be included in reports of observational studies

	Item No.	Recommendation	Page No.	Relevant text from manuscript
**Title and abstract**	1	(*a*) Indicate the study's design with a commonly used term in the title or the abstract	1	Line 1-2
		(*b*) Provide in the abstract an informative and balanced summary of what was done and what was found	3	Line 69-73
**Introduction**				
Background/rationale	2	Explain the scientific background and rationale for the investigation being reported	5	Line 79-102
Objectives	3	State specific objectives, including any prespecified hypotheses	6	Line 103-110
**Methods**				
Study design	4	Present key elements of study design early in the paper	6-11	Line 120-126, 201-221
Setting	5	Describe the setting, locations, and relevant dates, including periods of recruitment, exposure, follow-up, and data collection	6-11	Line 120-126, 201-221
Participants	6	(*a*) ***Case-control study***—Give the eligibility criteria, and the sources and methods of case ascertainment and control selection. Give the rationale for the choice of cases and controls	10	Line 213-216
		(*b*) ***Case-control study***—For matched studies, give matching criteria and the number of controls per case	10-11	Line 218-221
Variables	7	Clearly define all outcomes, exposures, predictors, potential confounders, and effect modifiers. Give diagnostic criteria, if applicable		
Data sources/ measurement	8*	For each variable of interest, give sources of data and details of methods of assessment (measurement). Describe comparability of assessment methods if there is more than one group	10	Line 210-216 + suppl. Methods
Bias	9	Describe any efforts to address potential sources of bias		n/a
Study size	10	Explain how the study size was arrived at		n/a

Continued on next page

Quantitative variables	11	Explain how quantitative variables were handled in the analyses. If applicable, describe which groupings were chosen and why		n/a
Statistical methods	12	(*a*) Describe all statistical methods, including those used to control for confounding	8-9	Line 169-176
		(*b*) Describe any methods used to examine subgroups and interactions		n/a
		(*c*) Explain how missing data were addressed		n/a
		(*d*) *Cohort study*—If applicable, explain how loss to follow-up was addressed<br>*Case-control study*—If applicable, explain how matching of cases and controls was addressed<br>*Cross-sectional study*—If applicable, describe analytical methods taking account of sampling strategy		n/a
		(*e*) Describe any sensitivity analyses		n/a
**Results**				
Participants	13*	(a) Report numbers of individuals at each stage of study—eg numbers potentially eligible, examined for eligibility, confirmed eligible, included in the study, completing follow-up, and analysed	16-17	Line 364-366
		(b) Give reasons for non-participation at each stage		n/a
		(c) Consider use of a flow diagram		n/a
Descriptive data	14*	(a) Give characteristics of study participants (eg demographic, clinical, social) and information on exposures and potential confounders		n/a
		(b) Indicate number of participants with missing data for each variable of interest		n/a
		(c) *Cohort study*—Summarise follow-up time (eg, average and total amount)		n/a
Outcome data	15*	*Cohort study*—Report numbers of outcome events or summary measures over time		n/a
		Case-control study—Report numbers in each exposure category, or summary measures of exposure		n/a
		Cross-sectional study—Report numbers of outcome events or summary measures		n/a
Main results	16	(*a*) Give unadjusted estimates and, if applicable, confounder-adjusted estimates and their precision (eg, 95% confidence interval). Make clear which confounders were adjusted for and why they were included		n/a
		(*b*) Report category boundaries when continuous variables were categorized		n/a
		(*c*) If relevant, consider translating estimates of relative risk into absolute risk for a meaningful time period		n/a

Continued on next page

Other analyses	17	Report other analyses done—eg analyses of subgroups and interactions, and sensitivity analyses		n/a
**Discussion**				
Key results	18	Summarise key results with reference to study objectives	18	Line 394-391
Limitations	19	Discuss limitations of the study, taking into account sources of potential bias or imprecision. Discuss both direction and magnitude of any potential bias		n/a
Interpretation	20	Give a cautious overall interpretation of results considering objectives, limitations, multiplicity of analyses, results from similar studies, and other relevant evidence	18	Line 403-412
Generalisability	21	Discuss the generalisability (external validity) of the study results	21	478-487
**Other information**				
Funding	22	Give the source of funding and the role of the funders for the present study and, if applicable, for the original study on which the present article is based	23	Line 511-526

*Give information separately for cases and controls in case-control studies and, if applicable, for exposed and unexposed groups in cohort and cross-sectional studies.

Note: An Explanation and Elaboration article discusses each checklist item and gives methodological background and published examples of transparent reporting. The STROBE checklist is best used in conjunction with this article (freely available on the Web sites of PLoS Medicine at http://www.plosmedicine.org/, Annals of Internal Medicine at http://www.annals.org/, and Epidemiology at http://www.epidem.com/). Information on the STROBE Initiative is available at www.strobe-statement.org.
